# Acoustic Manipulation of Intraocular Particles

**DOI:** 10.3390/mi13081362

**Published:** 2022-08-21

**Authors:** Ari Leshno, Avraham Kenigsberg, Heli Peleg-Levy, Silvia Piperno, Alon Skaat, Hagay Shpaisman

**Affiliations:** 1 Sheba Medical Center, Tel Hashomer, Ramat Gan 5262000, Israel; 2 Sackler Faculty of Medicine, Tel Aviv University, Tel Aviv 6997801, Israel; 3 Department of Chemistry and Institute of Nanotechnology and Advanced Materials, Bar-Ilan University, Ramat Gan 5290002, Israel

**Keywords:** acoustic manipulation, intraocular particles, standing waves, anterior chamber

## Abstract

Various conditions cause dispersions of particulate matter to circulate inside the anterior chamber of a human eye. These dispersed particles might reduce visual acuity or promote elevation of intraocular pressure (IOP), causing secondary complications such as particle related glaucoma, which is a major cause of blindness. Medical and surgical treatment options are available to manage these complications, yet preventive measures are not currently available. Conceptually, manipulating these dispersed particles in a way that reduces their negative impact could prevent these complications. However, as the eye is a closed system, manipulating dispersed particles in it is challenging. Standing acoustic waves have been previously shown to be a versatile tool for manipulation of bioparticles from nano-sized extracellular vesicles up to millimeter-sized organisms. Here we introduce for the first time a novel method utilizing standing acoustic waves to noninvasively manipulate intraocular particles inside the anterior chamber. Using a cylindrical acoustic resonator, we show ex vivo manipulation of pigmentary particles inside porcine eyes. We study the effect of wave intensity over time and rule out temperature changes that could damage tissues. Optical coherence tomography and histologic evaluations show no signs of damage or any other side effect that could be attributed to acoustic manipulation. Finally, we lay out a clear pathway to how this technique can be used as a non-invasive tool for preventing secondary glaucoma. This concept has the potential to control and arrange intraocular particles in specific locations without causing any damage to ocular tissue and allow aqueous humor normal outflow which is crucial for maintaining proper IOP levels.

## 1. Introduction

Aqueous humor (AH) is a clear low-viscosity fluid that contains a complex mixture of biological molecules secreted from plasma components by the ciliary body into the posterior chamber of the eye. From there the humor travels to the anterior chamber and proceeds to drain into the systemic cardiovascular circulation [1,2]. AH circulation is essential for the vitalization and protection of the surrounding non-vascularized tissues (trabecular meshwork (TM), lens, and corneal endothelium). Its secretion and outflow regulation are important physiological processes for the normal function of the eye [3]. In the healthy eye, the flow of AH against resistance generates the average intraocular pressure (IOP) necessary to maintain the proper shape and optical properties of the globe. Lack of AH transparency can cause decline in visual acuity, while reduction in its outflow can result in elevated IOP, which is a central source of several ocular pathologies, mainly glaucoma. Therefore, its synthesis, circulation, drainage, and consistency are of major clinical significance [1].

Various conditions can result in the formation or dispersion of particulate matter of different shapes, sizes, and characteristics that circulate into the AH. Table 1 summarizes the most common conditions and their corresponding pathological particles.

In addition to obscuring the visual axis in the acute phase, these particles can cause secondary complications, such as open-angle glaucoma, due to obstruction of the TM and the AH outflow [4,5,6]. Particles can also become deposited in the corneal endothelium, resulting in Krukenberg’s spindle or corneal staining from longstanding hyphema [7]. Although both medical and surgical treatment options are available for these complications, effective preventive options are lacking. As a result, current clinical practice takes mainly a “wait and see” approach, which involves treating only secondary complications and offering no treatment options for particulate matter disposal. In this study, we introduce a novel concept with potential clinical applications to manage and prevent complications in such conditions—acoustic manipulation, which can be used to control intraocular particles. The method precludes the need for surgical intervention and limits tissue interaction with particles by controlling their movement within the AH by acoustic radiation forces.

**Figure 1:**
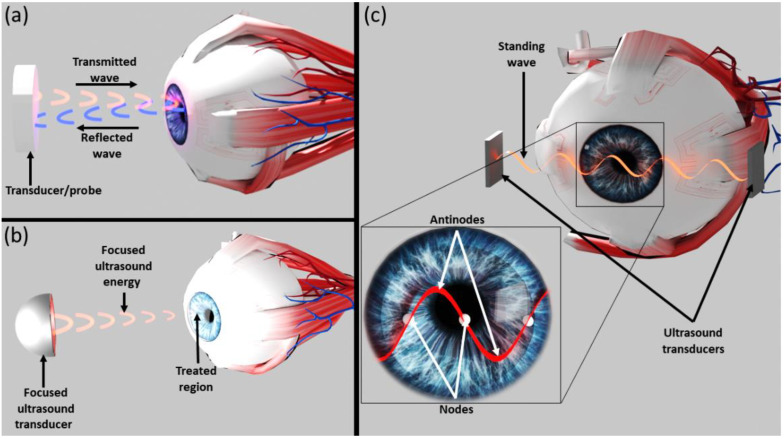
Illustration of various methods that utilize acoustic waves in ophthalmology. (**a**) Ultrasonic waves used to image the location and characteristics of various eye components. (**b**) High-intensity focused ultrasound used to destroy ocular tissue ablation. (**c**) The novel method for interocular particle acoustic manipulation suggested in this manuscript. Acoustic transducers are used to create standing acoustic waves that direct intraocular particles towards their nodal areas.

Sound waves have been used by physicians since the dawn of medicine, with auscultation as a fundamental part of the physical exam. In modern medicine, ultrasound, defined as sound waves with frequencies higher than the upper audible limit of human hearing, is the most common medical application of acoustics and is used mainly for imaging. In ophthalmology, imaging is achieved by an acoustic transducer that emits waves which are reflected from various components of the eye (Figure 1a). A probe (optionally the emitting transducer) detects the echoes and by measuring relative time and amplitudes can provide knowledge on location and characteristics of reflecting components. An array of transducers/probes is usually used, but for simplicity only a single element is illustrated in Figure 1a. The amplitude of the emitted waves can be increased to physically displace soft tissues while analyses of echoes is used for diagnosis [8] of the displaced tissue. Methods for retinal repair based on acoustic displacement have also been suggested [9].

Besides imaging, high-intensity focused ultrasound (HIFU) is used in ophthalmology due to its tissue-destructive properties. A focused ultrasound transducer emits waves that are focused to selected locations (Figure 1b). For example, HIFU is used to treat glaucoma by inducing selective and controlled thermic ablation of the distal part of the ciliary body [10]. Additionally, HIFU has been shown to induce hemolysis and short-term dispersion of a fresh blood mass in an intact eye [11].

In contrast to the above, here we explore a novel method of utilizing acoustic waves for ophthalmology. This is achieved by acoustically manipulating intraocular particles. Acoustic manipulation belongs to a family of methods that apply acoustic radiation forces to move particles. The phenomenon was first observed in 1831 by Faraday [12], who noted that light powder moves above a vibrating plate. When a dispersion of a fluid with solid particles is irradiated by a sound field, those particles scatter or absorb the acoustic field. Certain wave characteristics can move particles to specific space points where they can cluster. Among manipulation techniques such as electric, optical, optoelectronic, and magnetic tweezers, acoustic techniques have a significant advantage, as their only requirement is a difference in compressibility and density between the particles and surrounding medium (acoustic contrast), which is true for almost all dispersed systems. The power density required to manipulate particles with acoustic forces is also several orders of magnitude smaller than that of its optical counterparts and can influence a much larger area.

Acoustic waves have been used to manipulate and sort particles in 2D and 3D [13] to study particle–particle interactions, selectively promote coalescence [14], and create microstructures [15,16]. While manipulations of particles and biological cells have been observed before [17,18,19,20], to the best of our knowledge, the use of acoustic waves to control intraocular particles, rather than displace tissues as explained above [8,9], has not been described. An illustration of our new concept is presented in Figure 1c. Acoustic transducers are used to create standing acoustic waves that direct intraocular particles towards their nodal areas. Here we tested this concept experimentally to ensure that various aspects do not negatively affect the expected outcome. Such aspects include possible interactions between acoustic waves and biologic tissues that may disturb the wave pattern, and possible damage to the cornea due to undesired side effects that must be ruled out. The aim of this study was to determine the feasibility and safety of applying acoustic manipulation techniques to noninvasively control movement of particulate matter within the anterior chamber without causing damage to ocular tissues.

## 2. Materials and Methods

### 2.1. Acoustic Resonator

**Figure 2:**
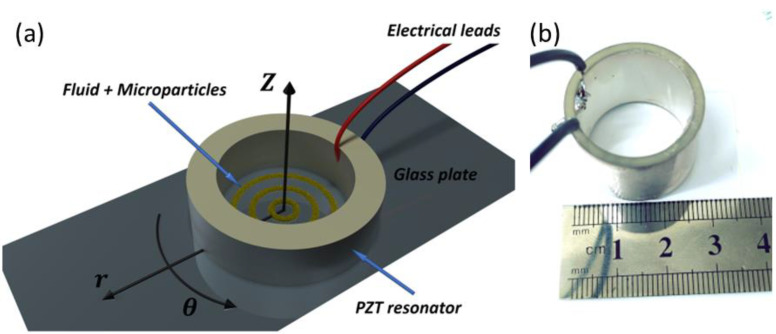
(**a**) Illustration (not to scale) of the experimental in vitro apparatus. The dispersed micro particles are inserted into the piezoelectric resonator. They are driven towards nodal areas and form rings upon activation of the acoustic waves. (**b**) Photograph of the in vitro apparatus.

Type-II (lead zirconate titanate (PZT)) acoustic radial resonators (inner diameter 22 mm, outer diameter 26 mm, length 20 mm) with a center resonance frequency of 1.1 ± 0.1 MHz were purchased from APC International (Figure 2a). The resonators were driven by a signal generator (Siglent, SDG 5162, Solon, OH, USA) with a continuous sine wave at an amplitude of 1–10 volt peak-to-peak (V_pp_) with 50 Ohm resistance to create pressure waves inside the reservoir. A power amplifier (X2, FYA2030, 3 MHz bandwidth with low distortion) was used for amplification to a maximum of 20 V_pp_. Experiments were conducted at 10–20 V_pp_, which translates to an effective transducer intensity of 20–80 mW/cm^2^ (power/vibration area).

To allow reproducibility using other acoustic resonators, we determined the speed and acoustic force applied on standard 10 µm diameter polystyrene beads (Alfa Aesar, Haverhill, MA, USA) in proximity to the pressure antinodes (where the acoustic field is maximal). Velocity was determined by tracking particle motion immediately after turning on the acoustic field (10 V_pp_), and was found to be 755 ± 159 µm/s after 0.3 s. The acoustic force can be expressed as F_acoustic_ = F_observed_ + F_drag_ and was determined to be 71 ± 15 pN.

A biocompatible soft coating was applied to the acoustic resonator to prevent direct contact with the eye: the resonator was first dipped in a Sylgard TM184 silicon elastomer base and then in a curing agent (Dow Corning, Midrand, MI, USA) mixed at a 10:1 weight ratio. Curing was performed for 2 h at 80 °C. The coating (polydimethylsiloxane) thickness was ~70 µm.

### 2.2. Particles

This feasibility study used two types of particles that represented pathologic and therapeutic particulate matter: (1) Pathologic particulate matter consisted of porcine pigment particles created by homogenization (D1000, Benchmark, 1 min operation with rotor speed set to 3000 RPM) of iris tissues retrieved from enucleated porcine eyes. Particle size ranged from ~50 µm to sub-micron particles. The density was not determined due to large variations in properties arising from nonuniform tissues used for their production. However, as they sediment over time, their density is >1 g/cm^3^. (2) Triamcinolone acetonide (TA; Triesence, 40 mg/mL, Alcon Std., Fort Worth, TX, USA) represented pharmacologic treatments as its milky appearance is easy to visualize in contrast to the dark brown iris of pig eyes. The average size of the TA particles was described previously to be 11.5 µm with a density of 1.3 ± 0.1 g/cm^3^ [21]. As seen in almost all solid particles immersed in liquid, both micro particles have a positive acoustic contrast factor in water and are expected to be driven towards the nodes of the acoustic wave.

### 2.3. In Vitro Model

**Figure 3:**
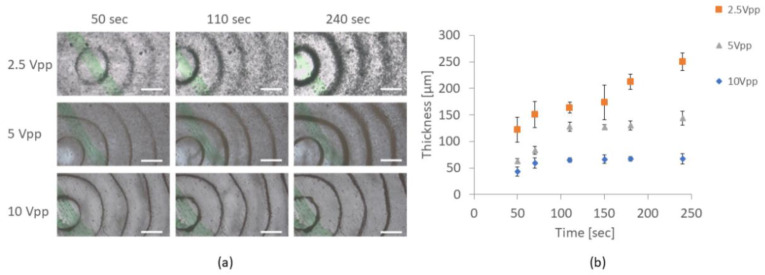
(**a**) Micrographs of triamcinolone acetonide particles arranged in concentric rings inside the acoustic resonator, corresponding to the location of standing acoustic wave nodes (spacing of 0.75 ± 0.1 mm). The thickness of the rings increased over time for all amplitudes as more material accumulated. Stronger acoustic waves resulted in thinner and denser rings. Scale bars = 0.7 mm. (**b**) Thickness of the second ring from the center as a function of time for various amplitudes.

An in vitro model was designed to evaluate and quantify the effect of the acoustic field on the micro particles. The piezoelectric cylinder was adhered to a glass plate (Figure 3) by high vacuum silicon grease (Dow Corning), and the reservoir was filled with 1.5 mL of deionized water containing 1.2 mg/mL of TA. An optical microscope (Eclipse LV-150, Nikon, Japan) with a CMOS camera (Deltapix HDMI16MDPX, Deltapix, Denmark) was positioned below the reservoir to capture and record pattern formations after activation of the acoustic wave. In order to quantify the acoustic effect, the widths of the ring structures were measured as a function of time at 50, 70, 110, 150, 180, and 240 s after activation. The analysis was performed on 4 different areas of the same ring (ring #2 from the center) for 3 wave amplitudes (2.5 V_pp_, 5 V_pp_, and 10 V_pp_).

### 2.4. Ex Vivo Model

Twelve enucleated porcine eyes were retrieved from the local abattoir within 4 h postmortem. For the purposes of the procedure, the eyes were fixed in place with a custom cut of 2 layers of Styrofoam. The piezoelectric resonator was placed securely on the eye, and normal saline solution was poured into it until the eye was completely covered in fluid.

The first six eyes were used for safety evaluation, two of which served as controls. Acoustic waves generated with 10 and 20 V_pp_ were applied for a duration of 1 and 5 min on individual eyes. Signs of possible corneal damage by acoustic energy absorption and thermal or mechanical damage were detected by the following methods: (1) Gross evaluation of changes via observation by an optical microscope (Eclipse LV-150N, Nikon, Japan); (2) a thermal imaging camera (Therm-App TH^®^, Opgal Optronic Industries Ltd., Carmiel, Israel) positioned above the apparatus to capture and record heat changes of the fluid reservoir after activation of acoustic waves, (3) Spectralis^®^ (Heidelberg Engineering, Heidelberg, Germany) optical coherence tomography (OCT) scans of the cornea and anterior segment obtained immediately after each procedure in order to detect signs of stromal changes of each eye, (4) after completion of the OCT scan, the eyes were immersed in 4% paraformaldehyde for 48 h and sent for histologic evaluation.

An additional six eyes were used to determine the effect of the acoustic wave on intraocular micro particles. Either TA or iris pigment particles were injected into the anterior segment in each eye by means of a clear corneal incision with a 30G needle prior to placement of the piezoelectric resonator. An optical microscope (Motic SMZ 171, Motic, China) with a CMOS camera (Moticam 580, Motic, China) was positioned above the apparatus to capture and record the pattern formations within the anterior chamber of the enucleated eye after activation of the acoustic wave.

## 3. Results

### 3.1. In Vitro

Without the acoustic waves, the TA particles that were dispersed in the cylindrical resonator sedimented over time and covered the glass slide in a homogeneous manner. Upon introduction of the acoustic waves, the particles formed concentric rings. Those rings corresponded to the location of standing acoustic wave nodes (discussed below) that were generally spaced apart at a mean distance of 0.75 ± 0.1 mm. As visualized qualitatively in the images presented in Figure 3, the thickness of the rings increased over time for all amplitudes as the TA particles sedimented over time (because of their higher density than water) and aggregated at the nodal areas. Stronger acoustic fields (higher amplitudes) resulted in narrower rings as the increase in acoustic force led to denser formation.

### 3.2. Ex Vivo

**Figure 4:**
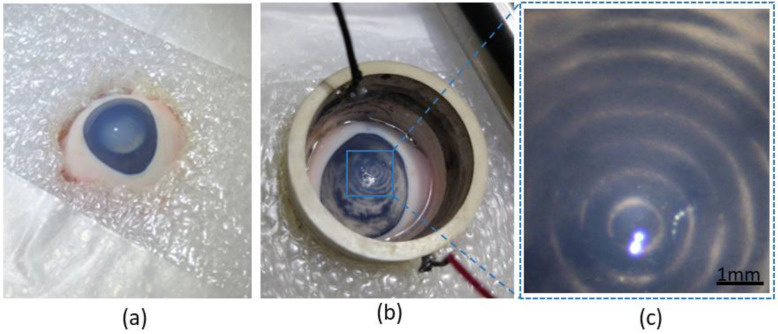
Ex vivo experimental setup: (**a**) fixation of an enucleated pig eye by means of two layers of Styrofoam. (**b**) After injection of the particles, the piezoelectric resonator was placed securely on the eye, and normal saline solution was poured inside until the eye was completely immersed in fluid. A ring arrangement of the triamcinolone acetonide particles within the anterior chamber can be seen. (**c**) Magnified microscope image showing the ring arrangement of porcine pigment particles within the anterior chamber.

Without acoustic waves, the particles underwent sedimentation over time to form a homogeneous layer over the iris and retina. Upon acoustic manipulation, both the pigment and TA particles that gathered in the anterior chamber became rearranged into concentric rings in a similar fashion to that observed in vitro when applying between 10 and 20 V_pp_ on the acoustic resonator (Figure 4), with variation only in the time required for arrangement. The pigment particles became arranged less quickly than the TA particles (Video S1 and Video S2, respectively) due to the smaller size of the former (discussed below). The concentric rings produced by the acoustic wave remained stable after its deactivation, with minimal changes after more than 1 h.

We attempted to further manipulate the particles by changing the frequency and physically moving the resonator. Slight variations in wave frequency around the resonance frequency (±100 kHz) produced slight changes in the arranged particles, while physically moving the resonator with respect to the eye resulted in movement of the particles towards the new location of the standing acoustic waves.

**Figure 5:**
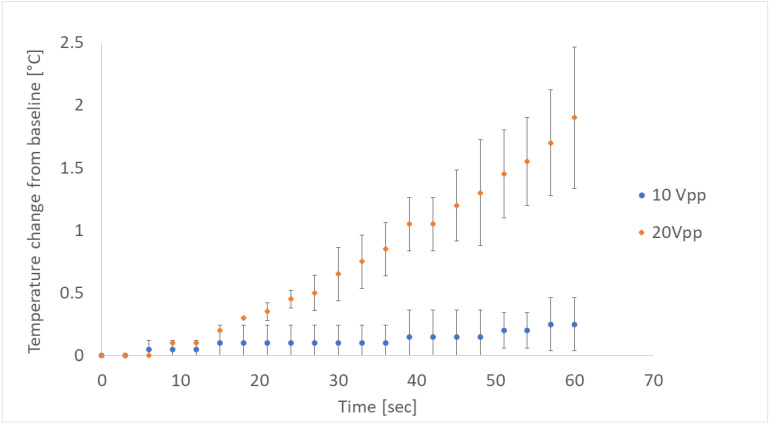
Temperature changes induced by the acoustic wave.

With regard to safety, neither macroscopic nor microscopic signs of tissue damage were observed during or immediately after activation of the acoustic wave, even for the strongest wave (20 V_pp_). Thermal imaging measurements of the fluid reservoir surface temperature showed almost undetectable changes when the acoustic wave was generated with a 10 V_pp_ signal, which resulted in a total increase of <0.5 °C after 60 s. When a 20 V_pp_ signal was applied, the fluid reservoir surface temperature rose at a relatively constant rate between 0.03–0.04 °C/s (Figure 5), yielding a total increase of 1.5–2.5 °C after 60 s. No gross signs of damage to the cornea, lens, or other anterior segment tissue were observed during the experiments.

**Figure 6:**
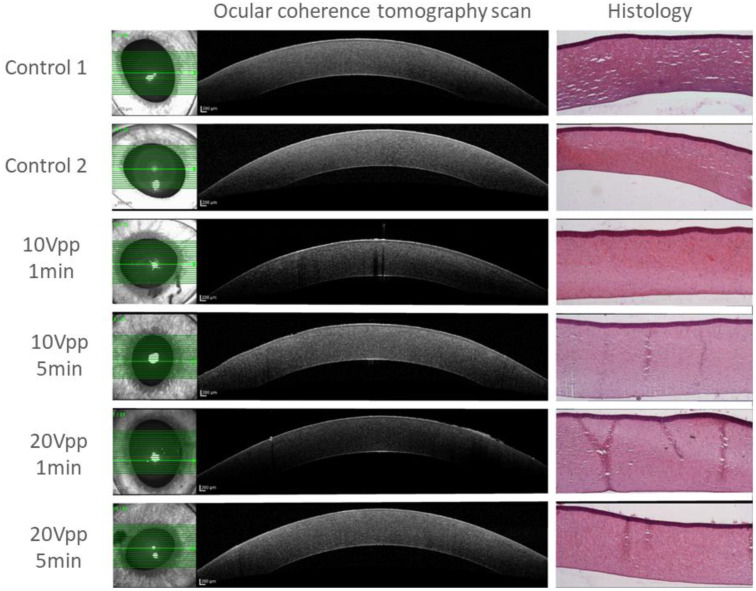
Post-treatment imaging and histology for various wave amplitudes and durations. No signs of adverse effects of the acoustic waves on the corneal endothelium or stroma compared to controls were detected.

The OCT scan of the anterior segment showed no signs of adverse effects of the acoustic waves on the corneal endothelium or stroma compared to controls (Figure 6). Some mild variations were observed between the eyes on the histology sections, which were determined by an experienced ophthalmic pathologist to be mere artifacts. The pathologist detected no sign of damage to the endothelium or stroma of the cornea, or any other side effect that could be attributed to the acoustic manipulation.

## 4. Discussion

Investigation of our novel method for ocular application of acoustic manipulation showed that acoustic forces can be harnessed for noninvasive manipulation of small particles within the anterior chamber, the applied energy had no injurious effect on the ocular tissues, and there were no apparent untoward side effects.

Acoustic manipulation is applied in various methods that enable particle movement by acoustic radiation forces. Our focus here was on the manipulation of particles by standing acoustic waves. There are positions along the standing wave (“nodes”) where the acoustic force is a constant zero at all times. Particles that are more dense and less compressible than the surrounding medium (thus having a positive acoustic contrast factor) will be driven to the nodal areas due to scattering of the standing acoustic wave (Figure 1c) [22]. This phenomenon may be conceptualized as the movement to “quiet” areas of particles that require more energy to vibrate in the acoustic field, thereby minimizing the total energy of the system.

Standing acoustic waves are widely used in the sorting, translating, rotating, and trapping of targets at the submicron and micron levels, especially particles and single cells. The technique enables manipulation of small as well as lightweight objects [23,24]. However, its use in medicine has been limited thus far. Ghanem et al. recently showed that it can be used transcutaneously to lift and reposition a stone in the urinary bladder of live pigs [24]. Here, we demonstrate the utilization of acoustic manipulation radiation forces to trap and manipulate the movement of intraocular particles. Such a novel noninvasive method could allow concentration of pathologic intraocular particles to a certain area where they would be unable to accumulate and obstruct the visual axis and intraocular structures (e.g., pseudoexfoliation and pigment dispersion) or cause visual disturbances (e.g., hyphema, asteroid hyalosis, and vitreous floaters). This is the first attempt to carry out noninvasive intraocular acoustic manipulation, which is a step forward following past works on larger organs [25]. The eye is considerably smaller and more delicate by comparison, and the particles being manipulated are ~3 orders of magnitude smaller; we observed here movement of particles <2 µm compared to 3 mm manipulated in pig urinary bladder.

Our study results show that such a form of intervention is feasible and safe. In the ex vivo model we were able to manipulate particle movement at 10 V_pp_ that translates to an effective transducer intensity of 20 mW/cm^2^ (power/vibration area), which is below the 50 mW/cm^2^ threshold for diagnostic ultrasound in ophthalmology determined by the US FDA [26]. Furthermore, even in the experiments conducted at 20 V_pp_ with an effective transducer intensity of 80 mW/cm^2^, we did not observe any sign of damage. Adverse effects on biologic tissues from the use of ultrasonic waves results mainly from thermal or mechanical damage, and the temperature changes observed in our experiments were very mild even at the highest energy levels. Although prolonged activation of the resonator might result in a more significant temperature rise, this can be easily managed by exchanging the fluid reservoir and cooling the system. Mechanical damage by cavitation bubbles was not detected, presumably because of the high frequency, which is far from the optimal conditions for effective cavitation impact [27].

Neither the histologic nor the OCT imaging evaluations yielded residual pathologic effect of the acoustic wave on corneal or other ocular tissues in the ex vivo study, suggesting that the acoustic waves are transmitted into the anterior chamber without causing structural damage. It also should be noted that the intensity in our experiments was approximately two orders of magnitude lower than interventional ultrasounds such as HIFU procedures used for glaucoma treatments [28].

**Figure 7:**
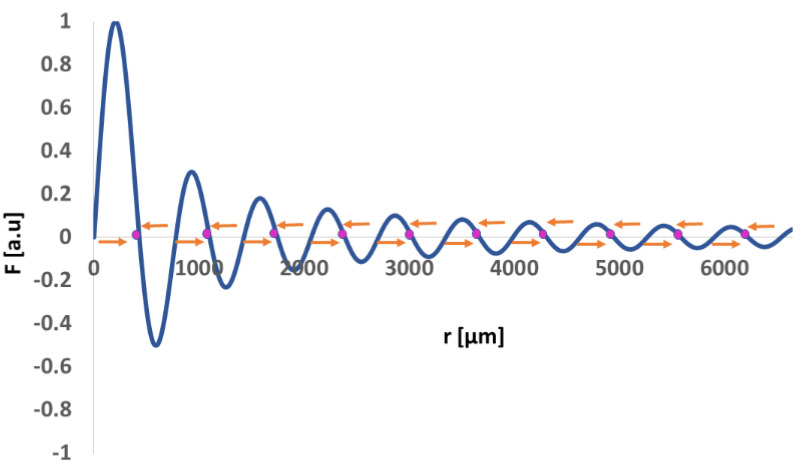
Variation in the normalized acoustic radiation force for a particle with a positive acoustic contrast factor along the radius of a cylindrical resonator, with r = 0 corresponding to the center of the resonator. The pink dots represent the nodal areas of the wave where an accumulation of triamcinolone acetonide particles was detected. The orange vectors represent the direction of the acoustic radiation force applied on micro particles.

The spacing between the concentric patterns was not constant across the diameter of the resonator. This is expected from a cylindrical reservoir. The dimensionless acoustic radiation force developed by Barmatz and Collas [29] is a rather complex concept of waves propagating in the radial direction under rigid boundary conditions. To simplify matters, we added an illustration (Figure 7) of the normalized acoustic radiation force for a particle with a positive acoustic contrast factor along the radius of a cylindrical reservoir (r = 0, corresponding to the center of the reservoir).

Indeed, we found accumulations of TA particles corresponding to the nodal areas of the wave where materials are expected to accumulate. The variations in distance between adjacent rings are rather small and therefore could be generalized as 0.75 ± 0.1 mm.

Given the positive dependence of particle volume on acoustic force [30], a relatively slower arrangement of the pigment particles compared to the TA particles is expected. According to bright field microscopy imaging, the former are considerably smaller than the latter, accounting for the variability in arrangement speed. Similarly, manipulation of even smaller particles could be achieved by allowing more time for arrangement. Arrangement into concentric circles was achieved in vitro for amplitudes in the range of 5 to 20 V_pp_. Higher amplitudes were positively correlated with speed and density of formed structures, as evident from the in vitro experiments. The amplitude of choice could, therefore, be tailored to a specific application. For example, various ocular conditions can result from the deposition or accumulation of pathologic particles in the anterior chamber where they interact with the intraocular tissues, causing obstruction of the visual axis, obstruction, and loss of function of the TM and drainage system, and subsequent IOP elevation. Treatment options for managing these particles and prevention of their side effects are currently lacking [31,32].

**Figure 8:**
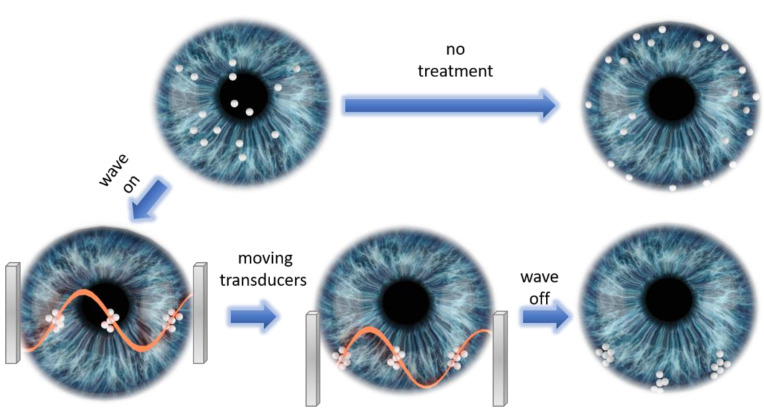
Illustration of suggested protocol utilizing acoustic manipulation for preventing secondary glaucoma. Without treatment, particles will flow towards the angle and distribute uniformly on the TM. Acoustically trapping particles at nodal areas followed by moving the acoustic transducers leads to placement of trapped particles at specific locations of the angle, thus leaving unaffected areas of TM that can support AH outflow.

The ability to noninvasively control movement of particles has several possible clinical applications in ophthalmology to help manage and treat such conditions. For instance, it can be used to clear the visual axis, enabling faster recovery and gain of function, in addition to clearing the field for a view of otherwise obscured pathologies and abnormalities. Moreover, it would be possible to protect the angle and TM from interaction with pathological material and prevent the development of secondary glaucoma. A conceptual illustration of such a process is shown in Figure 8. Interocular particles are initially spread throughout the anterior chamber, some of them obstructing the visual axis. Without any treatment, particles will eventually flow towards the angle and distribute uniformly on the TM. On the other hand, by utilizing our acoustic manipulation method one can selectively move particles to specific locations of the angle, thus leaving unaffected areas of TM that can support AH outflow. This could be achieved by applying the following steps: (a) trapping particles at nodal areas of the standing acoustic waves; (b) moving the acoustic transducers in respect to eye in such a way that the trapped particles will move to specific predefined angle locations; (c) turning off the acoustic waves, thus leaving the particles at the predefined locations.

This technique has other potential applications, for example: facilitating targeted treatment by directing intraocular medications to the appropriate site, assisting in the differential diagnosis of various intraocular particles, and possibly even assisting in the implantation of injected endothelial corneal cells by organizing them in a sheet-like pattern. In the future, it might even be possible to use it alongside robotic systems for surgery.

This study has several limitations. It shows that the technique not only causes the particles to organize in a specific pattern but also that the particle aggregate remains relatively stable. Because the models used were stationary, it was difficult at this point to determine the effect of eye motions on particle aggregates. Nevertheless, our experiments suggest that the effect of such aggregation will persist long enough to be clinically useful. Further in vivo experiments are needed to determine how long this aggregation will hold in order to determine the applicability of the technology in a clinical setting. We hypothesize that by moving the particles closer to the TM, the outflow of the aqueous humor will help fix them at their location and thereby protect the rest of the angle from the effects of the particles. Our safety evaluation focused mainly on the cornea and anterior segment, as they were most exposed to the acoustic energy in this study setup. While no signs of damage or adverse effects were found, we cannot completely rule out the possibility that some mild changes to the lens or posterior segment that might have been detected by a more detailed histologic evaluation were overlooked. Still, it is our opinion that the clinical significance of such findings would have been limited as we did not observe any significant changes of the anterior segment.

## 5. Conclusions

This study demonstrated that acoustic forces can be used to noninvasively manipulate movement of particulate matter within the anterior chamber without damaging the cornea. It is the first step in the development of ocular acoustic systems that will allow newer treatments and diagnostic capabilities for cases that at present are without treatment options. An important advantage of the technique is that the particles can be moved and controlled noninvasively, thus tempering the apprehension of complications of intraocular intervention. Further studies are required to explore the capabilities and possible ophthalmic uses of this novel methodology.

## 6. Patents

Leshno, A.; Skaat, A.; Kenigsberg, A.; Shpaisman, H. An Ocular Acoustic Device and a Method Thereof, PCT/IL2021/050862.

## Figures and Tables

**Table 1:** Pathologic conditions and their corresponding particulate matter.

Pathologic Condition	Particle Type
Pseudoexfoliation syndrome	Fibrillary extracellular material
Pigment dispersion syndrome	Pigment cells
Traumatic or spontaneous hyphema	Erythrocytes
Anterior uveitis	Leukocytes

## Data Availability

All data generated or analyzed during this study are included in this article. Further enquiries can be directed to the corresponding authors.

## Supplementary Materials

The following supporting information can be downloaded at: https://www.mdpi.com/article/10.3390/mi13081362/s1, Video S1: Ex vivo experiment showing pigment particle arrangement into concentric rings using acoustic waves emanating from a cylindrical resonator (10 V_pp_).; Video S2: Ex vivo experiment showing triamcinolone acetonide particle arrangement into concentric rings using acoustic waves emanating from a cylindrical resonator (10 V_pp_).

## Abbreviations

IOP	Intraocular pressure
AH	Aqueous humor
TM	Trabecular meshwork
HIFU	High-intensity focused ultrasound
PZT	lead zirconate titanate
V_pp_	Volt peak-to-peak
OCT	Optical coherence tomography
